# A One-Health evaluation of the burden of cystic echinococcosis and its prevention costs: Case study from a hypo-endemic area in Italy

**DOI:** 10.1016/j.onehlt.2021.100320

**Published:** 2021-08-30

**Authors:** Rudi Cassini, Massimo Canali, Francesca Tamarozzi, Andrea Angheben, Gioia Capelli, Federico Gobbi, Matteo Legnardi, Michele Brichese, Giuseppina Napoletano, Fabrizio Cestaro, Adriano Casulli, Michele Drigo, Maurizio Aragrande

**Affiliations:** a Dept. Animal Medicine, Production and Health, University of Padova, Viale dell'Università, 16, 35020 Legnaro, PD, Italy; b Dept. of Agricultural and Food Sciences, University of Bologna, Viale Giuseppe Fanin, 50, 40127 Bologna, Italy; c Dept. of Infectious-Tropical Diseases and Microbiology, IRCCS Sacro-Cuore Don Calabria Hospital, Via Don A Sempreboni, 5, 37024 Negrar di Valpolicella, VR, Italy; d Istituto Zooprofilattico Sperimentale delle Venezie, Viale dell'Università, 10, 35020 Legnaro, PD, Italy; e Veneto Region, Prevention and Public Health, Dorsoduro 3493, 30123 Venezia, Italy; f Azienda ULSS 9 Scaligera (Local Health Unit), Prevention Department, Via Valverde 42, 37122 Verona, Italy; g WHO Collaborating Centre for the Epidemiology, Detection and Control of Cystic and Alveolar Echinococcosis, Department of Infectious Diseases, Istituto Superiore di Sanità, Viale Regina Elena, 299, 00161 Roma, Italy; h European Union Reference Laboratory for Parasites, Department of Infectious Diseases, Istituto Superiore di Sanità, Viale Regina Elena, 299, 00161 Roma, Italy

**Keywords:** Cystic echinococcosis, Italy, One Health, Economic evaluation, Epidemiology

## Abstract

An integrated model, based on a One Health approach, was implemented to estimate the epidemiological and economic outcomes of cystic echinococcosis (CE) in Veneto region, an hypo-endemic area of Northern Italy, and the costs for its prevention. Data and information needed to populate the model were retrieved from published literature, official statistics, expert opinions, or actively searched through data mining (i.e., Hospital and slaughterhouse data), when fundamental data were not available. Human-health and animal-health costs, both public and private, were considered. The overall impact of CE in the study area was estimated in an yearly cost of about 0.5 million €, due to an average of 19.5 human hospitalized cases and about 200 infected animals among cattle and sheep, per year. The human:animal costs ratio was about 8:1. Most of the infected animals were autochthonous, while the identification of an autochthonous source of the infection for the human cases was extremely difficult, and unlikely in most cases. No specific action resulted to be in place for human surveillance, while veterinary surveillance accounted for a yearly cost of about 22,000 €. Sheepherders were found to pay privately an overall amount of around 2000 € for the preventive treatment of their dogs every year, but the applied protocol proved to be sub-optimal. The source of most of the human cases was likely external to the study area, and their economic impact accounts for a cost that is far exceeding that of surveillance and preventive actions in place in the veterinary sector. Although autochthonous human cases appeared to be very rare, the strengthening of preventive actions and surveillance systems can reduce the risk of their increment.

## Highlights

• The study integrated epidemiology and economic aspects in a One Health perspective.
• The research estimates in detail the overall impact of CE in a hypo-endemic region in Italy.
• The study provided data for evidence-based actions to control CE in the study area.
• The investigated case study represents a reproducible model for zoonotic diseases evaluation.

## 1 Introduction

Evaluation in the domain of health is often mono-disciplinary, and tends to disregard the complexity of drivers and outcomes belonging to different scientific domains. In recent years, the methodological approach for the evaluation of health measures and policies is changing toward a more comprehensive vision of health-related problems [1]. The One Health (OH) concept is a shift in this context, as it looks at the consequences of human actions in all the sectors involved in health, according to a cross-sectoral approach [2]. The OH approach requires inter-disciplinary competences to understand the complexity of the relationships between causes and effects. Evaluation also requires that the problem under analysis is an analytical unit (a system of relationships) relatively isolated from other systems.

This study represents the outcome of a research project aimed at evaluating public/private health interventions to control cystic echinococcosis (CE) in the Veneto region, North-Eastern Italy, to properly advise regional public health authorities. In Italy, regional health authorities have an important role in defining disease control programs and implementing surveillance activities. This project implemented an innovative evaluation methodology to investigate where policy strategies might be improved through a rational, cross-sectorial and interdisciplinary approach. The study area corresponded to the administrative territory of the Veneto region and CE was chosen as a case study, in consideration of its emerging nature in the area.

Veneto is a region of North-Eastern Italy covering 18,345 km^2^, of which 43.6% are hills and mountains. In 2019, the population was 4,907,704 inhabitants with an average Gross Domestic Product (GDP) per capita of 32,720 € (http://dati.istat.it/). The livestock sector is characterised by industrial farming, mainly for cattle (18,253 farms and 757,700 heads estimated in 2019) and swine production (2053 farms - 630,621 pigs in 2019), while forms of marginal and extensive husbandry dominate sheep and goat farming, with 73,359 and 26,336 heads estimated in 2019, respectively, and an overall number of 5831 farms (https://www.vetinfo.sanita.it/). The ovine farming also includes transhumant sheep flocks with 500–1000 adult animals per flock that move continuously across the region throughout the year. An overall amount of 60 flocks were recently estimated [3], although this figure is including some flocks moving to Veneto plains, but registered in neighbouring regions.

CE is a chronic and disabling disease in humans caused by the development of the larval stage of the cestode *Echinococcus granulosus* sensu *lato* (*s.l.*) [4]. In southern Europe, domestic ruminants (e.g. cattle and sheep) act as the main intermediate hosts for *E. granulosus s.l.*, and domestic dogs are the main definitive hosts. The disease is particularly widespread in areas where traditional sheep herding still represents an important source of income [5,6]. Indeed, this kind of sheep farming is common in most areas of Central, Southern and insular Italy, which are considered hyper-endemic or endemic [7]. On the contrary, Northern Italy, which is characterised by a more industrialized livestock sector, was historically considered free from the disease or with a hypoendemic/sporadic presence. However, the epidemiological features of CE have recently gained attention in Veneto. Recent literature demonstrated the circulation of the parasite in different animal populations [8] and reported records of human cases [9]; however the connection between circulation of the parasite in the animal populations and transmission risk to humans has not been investigated so far.

## 2 Materials and methods

### 2.1 Model building

**Fig. 1:**
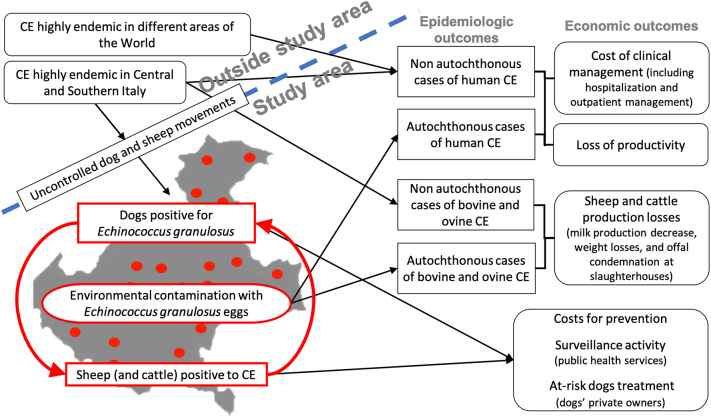
Epidemiologic-economic model flowchart of cystic echinococcosis in Veneto (modified from [10]).

An integrated epidemiologic and economic model (EEM) and a data collection strategy were developed to better define, at regional level, the main characteristics of CE in the context of human, animal and environmental health. The EEM was based on literature review and expert consultation, using an interdisciplinary approach reproducible for other contexts and diseases [10]. Briefly, the EEM was constructed thanks to the interaction between the research team (RT), composed by researchers in economics, human health and animal health, and an interdisciplinary network (IDN) of professionals and scientists operating as an external advisory board, to validate the main outcomes of the model. The IDN members have been selected considering the integrative expertise needed to widen the RT institutional and operational perspective, in critical aspects of public health management, veterinary services implementation and One Health research. The first tentative model was submitted by the RT to the IDN members and the revised version was discussed and agreed between the RT and the IDN in a one-day meeting [10]. The model investigated the interrelations between epidemiology and health economics: the distribution and intensity of the infection in animals and humans could be evaluated in economic terms by joining the disease epidemiology with the economic functions. These interrelations were visualised using a flow chart (Fig. 1).

The EEM was built to allow the identification of CE costs in the region and the type of data needed to develop the OH evaluation. The data to be collected were identified through the interactive process between RT and IDN and consisted in i) the number and anamnestic data of human CE cases; ii) the cost for clinical management and loss of productivity of human cases; iii) the number of sheep and bovines found infected at slaughterhouse; iv) the costs related to offal condemnation and decreased productivity; v) the costs for preventive measures in animals and humans.

### 2.2 Data collection methodology

#### 2.2.1 Number and distribution of human cases

The number of hospitalized human CE cases was obtained from Hospital Discharge Records (HDR), based on a dataset provided by the Italian Ministry of Health (MoH - protocol n. 23,875, date 06/08/2018) (Supplementary Material S1). All individuals, born in Italy or abroad, residing in Veneto and hospitalized in Italy between 2005 and 2016 with primary or non-primary diagnosis of echinococcosis (Codes 1220–1229 of the International Classification of Diseases - Version 9 - Clinical Modification – ICD9CM) were included in the dataset [11]. Anonymized data on nationality, sex, age, living place (Municipality and Province), number of days of hospitalization, ICD9CM code, cyst localization, and type of clinical management (medical or surgical) of each admission were provided by the MoH and elaborated to display a simple statistical description of case distribution. Patients with multiple admissions were considered only once; furthermore, when different clinical managements (surgical and medical) were applied in different admissions, the patient was considered as only treated surgically. It was not possible to obtain data on CE cases diagnosed and managed as outpatients. CE in Italy is a notifiable disease not requiring single case reporting but only annual transmission of summary figures (number of cases seen in the year) to the regional health authorities which in turn should transmit these figures to the national health authority and from here at European level [12]. Unfortunately, this reporting system is not properly implemented; therefore, de facto, the only available data on human CE in Italy come from hospitalized cases that are captured automatically by the HDR system.

Autochthonous and non-autochthonous cases were differentiated mainly based on birth country and its endemicity for CE. For Italian patients, only young individuals (<30 years of age) living in a Municipality included in a known cluster of bovine CE [8,13] were considered as suspected autochthonous cases.

#### 2.2.2 Cost of human cases

The estimation of the economic impact of human CE was based on the evaluation of clinical management costs and productivity losses.

Three types of relevant primary diagnosis of “echinococcosis” and respective clinical management protocols were considered: surgical treatment of hepatic echinococcosis; surgical treatment of thoracic echinococcosis; and hospital-based medical treatment of both hepatic and thoracic echinococcosis. As albendazole treatment does not require hospitalization, it can be speculated that these hospitalized but only medically treated cases represent patients with CE hospitalized for the purpose of in-depth diagnostic procedures (differential diagnosis, evaluation of treatment options, etc). For non-primary diagnosis of echinococcosis, it was possible to associate CE cases to a specific hospitalization cost if surgically treated, while solely the pre- and post-hospitalization costs were considered for the medically treated cases. The cases with undefined localization were attributed to hepatic or thoracic echinococcosis, in the same proportion as in the whole dataset. Hospitalization costs were deducted from the standard costs set by the Regional Health Authority for each type of diagnosis, identified by a specific Diagnosis Related Group (DRG) code [14]. By integrating hospitalization costs with pre- and post-hospitalization medical procedures, we defined standard procedures for each relevant type of treatment and assigned a unit cost to each step of the process. Clinicians from the reference hospital for Tropical Diseases in Veneto (Sacro Cuore Don Calabria, Negrar, Verona), involved in the management of patients with CE, were asked to delineate common features of the clinical management of patients undergoing different treatment procedure, from the diagnostic process to the follow-up. Features requested to the experts included types of exams prescribed for the diagnosis of CE (e.g. serology, imaging); dose and length of drug prescription in relation with the defined medical or surgical management; and type and frequency of exams to be performed during the recommended treatment and follow-up (e.g. imaging, blood test to monitor side effects of the drugs). The clinicians involved in the definition of the standard procedures apply in their setting the recommendations of the WHO Informal Working Group on Echinococcosis (WHO-IWGE) [15]. Standard procedures allowed the identification of costs for health care services supported by public institutions and private individuals, and patients' productivity losses. The costs for health care services supported by public institutions correspond to the reimbursements paid by the regional administration to hospitals and laboratories delivering such services. Costs for health care services supported by private individuals were the patients' out-of-pocket expenditures integrating the regional payments for some medical services (i.e. participation in the cost of some analyses; cost of drugs not reimbursed by the public health administration). The patients' loss of productivity consists in the time lost for medical visits, hospitalization, and convalescence. Cost calculation applied the transformation of the current available monetary data into 2019 euros (€_2019_). We adopted standard costs established by the Veneto Region in 2011 [14], then translated in the corresponding values of 2019 by applying the national consumer price index (Italian national Institute for Statistics - ISTAT, https://www.istat.it/it/archivio/30440). All values were provided by the Sacro Cuore Don Calabria hospital administration (reference centre for Tropical Diseases in Veneto) and established by the regional law [14], also concerning the contribution requested from private individuals.

The unit cost of productivity losses due to human CE was calculated by assigning different percentages of temporary disability according to the type of disease management (surgical or medical). Disability weights and their duration (up to 5 years) for abdominal and thoracic CE were deduced from the available literature [16,17], converted into working time losses, and then into monetary values. In details, a disability weight of 0.8 for the first month, 0.2 for the first year [16] and 0.1 for the following four years [17] were used for surgical interventions, while disability weights of 0.1 for the first six months and 0.01 for the first five years [17] were adopted for medical management. Productivity losses were estimated by multiplying the above-mentioned rates (disability weights - DISw) for the GDP/inhabitant/working day (GDPwd) and for the number of days (WDav) lost because of the illness (for each of the identified sickness periods), according to the formula: productivity losses = DISw*WDav* GDPwd. The GDPwd was obtained dividing the average GDP/inhabitant in the Veneto Region during 2005–2016 (GDPav) by the average number of working days during 2005–2016. The GDPav was calculated transforming the annual regional GDP/inhabitant into €_2019_ value, and multiplying the annual data by the corresponding monetary transformation index (both data available at the ISTAT website, resulting respectively from on line queries and historical tables of monetary transformation indexes - http://dati.istat.it/) and calculating the average of this new €_2019_ series. The annual WDav resulted from the average of the annual working days during the considered period. Annual working days are available at https://api.workingdays.org/1.2/api-countries.php. The number of annual working days is rather stable during the period (average: 253, min÷max: 250÷254). The number of WDay for each considered periods were calculated dividing the annual average by the appropriate rate (e.g. one month Wday = annual Wday/12).

#### 2.2.3 Number and distribution of cases in livestock

The number of slaughtered bovines and sheep in the Veneto region were obtained from the veterinary National Information System (https://www.vetinfo.sanita.it/) for the years 2014–2017 (Table 1 and Supplementary Material S3).

**Table 1:** Parameters used in the model to calculate the costs for animal component. Parameters obtained from official statistics and data mining were calculated using the period 2014–2017.

Input parameter	Abbreviation in formulas	Average value	Interval		Description of lower and upper values calculation	Ratio of difference between upper and lower values compared to the average	Source of data
			Lower	Upper			
CE prevelence in dairy cows	CE*bprev*	0.17%	0.16%	0.19%	avg + −2*SE	0,18	[13]^1^
CE prevalence in sheep	C*Eoprev*	6.05%	5.1%	7.0%	avg + −2*SE	0,31	This study - data mining
N slaughtered dairy cattle	N*bsl*	59,355	54,488	64,222	avg + −2*SD	0,16	National Information System^2^
N slaughtered adult sheep	N*osl*	2574	917	4231	avg + −2*SD	1,29	National Information System^2^
Price of bovine liver	P*bl*	2.83 €/kg	2.38 €/kg	3.28 €/kg	Provided in the source of data	0,32	Commercial Chamber^3^
Cost of offal disposal	K*disp*	0.44 €/kg	0.44 €/kg	0.44 €/kg	Fixed value	0	[19]
Price of raw milk	P*milk*	40.36 €/hl	34.05 €/hl	46.68 €/hl	avg + −2*SD	0,31	Verona market, Veneto^4^
Price of sheep meat	P*meat*	1.02 €/kg	0.97 €/kg	1.07 €/kg	Provided in the source of data	0,10	Commercial Chamber^5^
Bovine liver weight	W*bli*	5.86 kg	4.18 kg	7.54 kg	avg + −2*SD	0,57	[18]^6^
Bovine lungs weight	W*blu*	3.34 Kg	2.18 kg	5.50 kg	avg + −2*SD	0,99	[18]^6^
Reduction in milk production in bovines	L*milk*	7.3%	2.5%	12.0%	Provided in the source of data	1,30	[21]
Milk production in Veneto Region	Q*milk*	9,936,731 hl	9,458,398 hl	10,415,064 hl	avg + −2*SD	0,10	ISTAT^7^, ^8^
Ovine lungs weight	W*olu*	0.72 Kg	0.72 Kg	0.72 Kg	Fixed value	0	[20]^9^
Ovine liver weight	W*oli*	0.45 Kg	0.45 Kg	0.45 Kg	Fixed value	0	[20]^9^
Reduction in growth in ovine	L*meat*	11.3%	2.5%	20.0%	Provided in the source of data	1,55	[21]
Dead weight of adult sheep carcass	Q*meat*	27.2 kg	22.0 kg	32.4 kg	avg + −2*SD	0,35	ISTAT^7^

1 Corrected considering an estimated denominator of 266,880 (142,488 × 1.873). 142,488 is the denominator used in [13] and 1.873 is the by the ratio between total slaughtered dairy cattle and that slaughtered in Veneto, according to data provided by the National Information System.

2 https://www.vetinfo.sanita.it/

3 https://www.to.camcom.it/

4 https://www.clal.it/

5 https://www.fe.camcom.it/

6 transformed according to ratio 1 kg = 0.4536pound.

7 http://dati.istat.it/

8 In hectolitres, using 0.971 as conversion rate from 100 Kg (q) to 100 l (hl)

9 Variability estimates not provided.

To estimate the number of infected bovines we relied on the infection rate calculated in a previous 5-years investigation, which was considering only dairy cows, since beef calves and bulls showed an irrelevant number of cases [13]. A correction was applied to the prevalence estimate, since it was calculated based on ISTAT data (http://dati.istat.it/), which consider only the animals slaughtered in the Region and do not include the Veneto's livestock slaughtered outside the Region. Based on this calculation, the value (0.33%) reported in [13], was converted in a prevalence estimation of 0.17% (95%CI: 0.16–0.19%) (Table 1).

Since there was no available data concerning CE prevalence in sheep, the number of adult sheep with echinococcal cysts was estimated through a survey conducted in a selected group of slaughterhouses. All the units slaughtering more than 100 adult sheep yearly were contacted to obtain the estimated infection rate of sheep at inspection. Totally, 11 sheep slaughterhouses (5 outside and 6 in Veneto) were contacted and 7 replied (of which 3 in Veneto), providing data for one or more years (see S3 for details). Only adult sheep (>1 year) were considered, whereas younger sheep were not included in the analysis, since the data for this category were provided in an aggregated form (slaughtered lambs coming from a single farm were registered collectively as a group, without specifying their number). However, these animals have generally a very low prevalence of infection, due to the low probability of acquiring the infection and presenting a developed CE cyst within the first months of life.

#### 2.2.4 Cost for offal disposal and loss of productivity in animals

Offal disposal and productivity losses of infected animals were considered for the estimation of the economic impact of CE on animal production.

The economic loss for offal condemnation is the foregone income due to unsold products and cost of organ destruction, which has to follow specific protocols, if the organ is declared infected. As per the foregone income of infected organs at slaughter, only bovine livers were considered, since bovine and ovine lungs and ovine livers have little or no economic value in the study area. Unit values for organ weights and relevant costs were retrieved from available literature [[18], [19], [20]] and official statistics. The available monetary data were transformed into €_2019_ values (Table 1).

The calculation of the economic impact on animal productivity included the losses from reduced weight gain in sheep and reduced milk production in cows. Indeed, milk-producing sheep in Veneto represented only the 2.7% (1966/73,359) of the total sheep heads in the 2019 in Italy (https://www.vetinfo.sanita.it/), whereas almost all affected bovines were dairy cows. Historical series of the amount and prices of livestock production in Veneto were obtained from official statistics (Table 1). Indicators of productivity losses, were deduced from the available literature [21]. The average/intermediate value, the uncertainty and the source of all parameters used in the model for animal costs are reported in Table 1 and the formulas to calculate the overall costs for offal disposal and loss of productivity are reported in Table 2.

**Table 2:** Formulas to calculate the costs for offal disposal and loss of productivity (all formulas refer to one-year timespan).

Costs	Cattle	Sheep
Foregone income due to unsold livers	(1) K_B1_ = N*bsl*_⁎_ CE*bprev*_⁎_ W*bli*_⁎_ P*bl*	–
Cost of organ destruction	(2) K_B2_ = (W*bli* + W*blu*) _⁎_ K*disp*_⁎_ N*bsl*_⁎_ CE*bprev*	(4) K_O1_ = (W*oli* + W*olu*) _⁎_ K*disp*_⁎_ N*osl*_⁎_ CE*oprev*
Loss in milk production	(3) K_B3_ = Q*milk*_⁎_ CE*bprev*_⁎_ L*milk*_⁎_ P*milk*	–
Loss due to reduced growth	–	(5) K_O2_ = Q*meat*_⁎_ CE*oprev*_⁎_ L*meat*_⁎_ P*meat*
Notes	N*bsl* = nr of slaughtered dairy cows W*bli* = weight of bovine liver (kg) P*bl* = price of bovine liver (€/kg) W*blu* = weight of bovine lung (kg) K*disp* = unit cost of disposal (€/kilo) Q*milk* = yearly bovine milk production (hl) CE*bprev* = prevalence of CE in dairy cows (%) L*milk* = loss of milk production in infected cattle (hl) P*milk* = price of bovine milk (€/q)	N*osl* = nr of slaughtered adult sheep W*oli* = weight of ovine liver (kg) W*olu* = weight of ovine lung (kg) K*disp* = unit cost of disposal (€/kilo) Q*meat* = weight of ovine carcasses (kg) CE*oprev* = prevalence of CE in sheep (%) L*meat* = loss of meat production in infected sheep (kg) P*meat* = price of sheep meat (€/kg)

#### 2.2.5 Actions in place to prevent infection spread

**Fig. 2:**
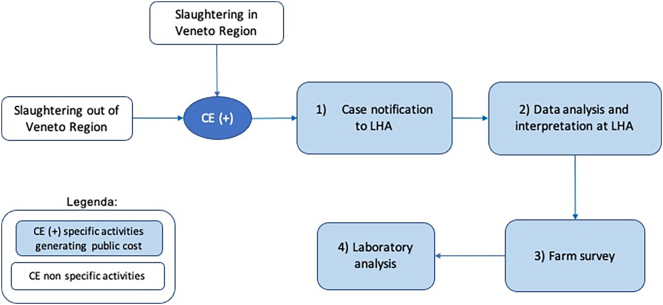
Flow diagram of the procedure in place for veterinary surveillance of CE in the Veneto region. CE = cystic echinococcosis. LHA = Local Health Authorities.

The estimation of the overall costs for preventive health measures included the resources spent for veterinary surveillance activities and for the periodic treatment of dogs at risk of infection. Regarding veterinary surveillance, no structured official plan is adopted by the Region: passive surveillance procedures, based on the general indications of Directive 2003/99/EC, are implemented by the nine Local Health Authorities (LHAs) operating in the Veneto region. A structured questionnaire was submitted to veterinarians in charge of CE surveillance in all LHAs to obtain information on the current procedures for the management of bovine and ovine cases in the Region, and time allocation for each phase of the procedure. The answers received by eight of them were summarized in a flow diagram (Fig. 2) and in the related description of each phase of the procedure. Cost estimation was based on standard costs for veterinary services provided by LHAs [22] and on standard costs applied to laboratory analyses by the Regional Laboratory in charge of animal health surveillance, i.e. the Istituto Zooprofilattico Sperimentale delle Venezie (IZSVe - https://www.izsvenezie.it). All costs were converted in €_2019_.

The estimation of the costs for the periodic treatment of dogs was aimed at identifying the overall costs for the hypothetic scenario of an appropriate treatment protocol applied to all dogs at risk, and the real scenario of the treatments actually in place. Sheep dogs were identified as the dogs at risk of infection, and their number was obtained thanks to relevant literature [3,8]. The canine population was estimated in 258 sheep dogs, based on the presence of about 60 transhumant sheep flocks living in or passing throughout the region [3] and an average number of 4.3 dogs per flock [8].

An average market price for praziquantel-based drug available in the Italian market was estimated based on information provided by a retail franchise of pet supplies. In consideration of the high variability in weights among dogs, the average dose was assumed to correspond to that for a 15–20 kg dog.

To calculate the overall cost for both scenarios, the price of a treatment with anthelmintic drug (i.e. praziquantel) was multiplied by the number of administrations performed in a time unit (one year), and by the estimated number of treated dogs in Veneto, according to the following formula:

(6) $$K\mathrm{dog}=P{\mathrm{admin}}_{*}\;N{\mathrm{admin}}_{*}\;N\mathrm{dog}$$

where:

K*dog* = overall yearly cost of dogs' preventive treatment.
P*admin* = average price of one administration of appropriate drug (€_2019_/dose).
N*admin* = number of administrations per year.
N*dog* = number of dogs treated.

Regarding active surveillance (e.g. ultrasound surveys) and preventive measures in humans (e.g. specific health education campaigns), these were not included in the EEM, as no such activities are implemented in the study area.

### 2.3 Uncertainty and data synthesis

To account for the uncertainty, the intermediate estimates, the lower estimates (best-case scenario) and the upper estimates (worst-case scenario) of each parameter were included in the model and displayed, whenever possible. The 95%CI lower and upper values of the parameter distribution were considered as the lower and upper estimates (i.e. calculated prevalence ±2*Standard Error for sample-based prevalence values; average ± 2*Standard Deviation for quantitative parameters). In some cases, parameters were considered fixed, if determined by local legislative acts (e.g. for medical costs supported by the regional health system), or if based on literature data, when the published paper was not reporting any uncertainty value. The number of hospitalized human cases was also considered as actual and certain, but the interannual variability was taken into account to determine the variability surrounding the estimation of their annual costs.

In the final step of data analysis, all collected data were summarized in a new flow chart to visualise the different contributions each component was bringing to the overall burden of the disease, and their relative uncertainty interval. This exercise was aimed at supporting decision making for cost-effective health interventions.

## 3 Results

### 3.1 Impact of CE on human health

A total of 234 residents of the Veneto region (90 Italian-born and 144 foreign-born) were admitted to hospitals and treated for CE in the study period, with a mean of 19.5 cases per year (95%CI: 9.1–29.9) and without any clear decreasing or increasing trend over time. Considering the total population of the Region as population at risk, we could therefore estimate an incidence rate of hospitalized cases of 0.4 cases/100,000 people per year (95%CI: 0.2–0.6). The cases were similarly distributed between sexes, with 121 males (52%) and 113 females (48%). Only 8% (19/234) were young individuals (≤20 years), the majority (166/234, 71%) were aged between 21 and 60 years, and the remaining (49/234, 21%) were over 60 years of age. Some cases were recorded as alveolar echinococcosis (ICD9CM 1225–1227). However, in Italy only CE is endemic, while no autochthonous or imported cases of human alveolar echinococcosis (AE) are currently known [23]. Based on the epidemiological situation of AE in the countries of origin of these patients that is absent or extremely rare [24], and on the highly unlikely fact that a true diagnosis of AE in Veneto would have been posed without the assistance for its diagnosis and complex clinical management of the regional referral centre, we considered that they were CE cases with multi-loculated cyst appearance, e.g. 2CE or CE3b stages of the WHO-IWGE classification [15]. The distribution of human cases according to cyst localisation and type of clinical management is reported in Table 3 (S1 for details).

**Table 3:** Number of hospitalized human CE cases according to localization of the cyst (undefined, liver, lung) and type of management (surgical, medical) in the Veneto region in the period 2005–2016.

Diagnosis	Localisation	Type of clinical management		Total
		Surgical	Medical	
Primary	Undefined	17	16	33
	Liver	99	37	136
	Lung	4	4	8
Non-primary	Undefined	1	16	17
	Liver	8	31	39
	Lung		1	1
	Total	129	105	234

The majority of cases (129/234, 55%) were treated surgically, whereas 105 patients (45%) received medical treatment as sole intervention. For 177/234 (76%) cases, CE was a primary diagnosis. Considering only cases with a single hospitalization (*n* = 136), the estimated mean number of days of hospitalization was 13.8 days for surgical and 10.9 days for medical management.

Standard DRG reimbursement for one hospitalized CE case ranged from 2793 to 10,736 €_2019_ depending on cyst localisation (liver or lung) and type of treatment. However, including pre- and post-hospitalization and loss of productivity, a single human CE case would cost between a minimum 6959.84 €_2019_ and a maximum 35,354.98 €_2019_ (Table 4 and S2 for details). When considering all cases recorded as CE-related HDR (S2 for details), the total cost for the Veneto region in the period 2005–2016 was estimated to reach 5,313,506.13 €_2019_ (4,392,292.55 €_2019_ for surgical treatments and 920,213.58 €_2019_ for medical treatments), for a yearly mean of 442,792.18 €_2019_ (95%CI: 170,074.32-715,510.03 €_2019_).

**Table 4:** Cost structure of CE clinical management protocols in Veneto region (€_2019_).

Description of clinical management protocol (DRG code)	Hospitalization cost	Pre- and post-hospitalization costs		Loss of productivity	Total costs
	(public)	(public)	(private)		
Abdominal surgery of liver CE (DRG 192)	8464.29	665.86	393.97	24,583.51	34,107.63
Thoracic surgery of lung CE (DRG 075)	10,736.06	181.15	242.62	24,195.15	35,354.98
Medical treatment of CE - primary diagnosis (DRG 423)	2792.57	1167.69	707.80	5615.11	10,283.18
Surgical treatment of CE - non-primary diagnosis (DRG 468)	7083.20	665.86	393.97	24,583.51	32,726.54
Medical treatment of CE - non-primary diagnosis	–	1167.69	707.80	5084.35	6959.84

The estimate of the relative percentage of autochthonous and non-autochthonous human CE cases was difficult to achieve. Foreign-born patients (144/234, 61.5%) were mostly from countries endemic for CE (e.g. Morocco = 55; Romania = 29; Moldova = 14; North Macedonia = 8; Tunisia = 7) and therefore we assumed that the infection was most likely acquired in their country of origin. Concerning Italian-born patients (90/234, 38.5%), our data did not allow understanding if patients were born in Veneto or in other Italian regions (possibly endemic for CE) and for how long they had been residing in Veneto. Out of 5 persons under 30 years of age, it was possible to identify only one case, a 20-year old female living in a municipality included in a cluster of bovine CE identified in 2014 [8], who we classified as autochthonous case.

### 3.2 Impact of CE on animal health

An average of 59,355 dairy cows (95%CI: 54,488-64,222) raised in the region were slaughtered yearly in the period 2014–2017. Considering an estimated prevalence of 0.17%, 101 infected animals (95%CI: 87–122) were estimated to be found in the region each year. As per ovines, an average of 2574 adult sheep (95%CI: 917–4231) belonging to farms based in Veneto were slaughtered yearly in the period 2014–2017. The estimated infection rate of CE in slaughtered sheep was 6.0% (CI95% 5.1%–7.0%). Consequently, an overall average of 156 sheep (95%CI: 47–296) were estimated to be infected on a yearly basis (see S3 for details).

The overall cost for offal condemnation and production losses were calculated according to Table 1 parameters and Table 2 formulas (see S3 for details). An average annual economic impact of 53,282.75 €_2019_ (lower estimate: 14,345.45; upper estimate: 119,312.26) was estimated, mostly attributable to loss in milk production from infected dairy cows (Table 5).

**Table 5:** Yearly average number of infected animals and consequent yearly economic losses due to CE in cattle and sheep (€_2019_).

	Infected animals (N)		Costs (€_2019_)								Total	
			Foregone income due to unsold livers		Cost of organ destruction		Loss in milk production		Loss due to reduced growth			
	Average		Average		Average		Average		Average		Average	
	Lower	Upper	Lower	Upper	Lower	Upper	Lower	Upper	Lower	Upper	Lower	Upper
Cattle	**101**		1673.36		408.46		50,634.74				**52,716.56**	
	87	122	867.66	3.017.06	243.99	646.40	13,184.76	113,442.71			14,296.40	117,106.17
Sheep	**156**				80.09				486,10		**566.19**	
	47	296			24.08	152.48			24.96	2053.62	49.04	2206.09
Overall											**53,282.75**	
											14,345.45	119,312.26

As per definition of the percentage of autochthonous cases, the movements tracking system in place for sheep did not allow for a certain identification of the most likely origin of the infection, although most sheep raised in Veneto should be considered locally born, according to expert opinion. Concerning bovines, according to previous data [13], 81.1% of cattle found positive for CE in Veneto were autochthonous.

### 3.3 Cost of preventive measures in place

The costs of the procedures currently in place in the Region for veterinary surveillance (flow diagram in Fig. 1) were calculated for bovine farms, whereas LHAs reported that sheep CE cases are never or rarely notified. A description of the activities, time allocation and associated costs of the four procedural steps of the surveillance procedure are shown in Table 6.

**Table 6:** Details of cost estimation of the four steps of veterinary surveillance procedure for the management of a bovine CE case (€_2019_).

Step	Description	Activity	Resource	Unit resource allocation^1^	Full cost (€_2019_)	Implementation rate	Average cost (€_2019_)
1	CE case notification at slaughterhouse	CE(+) case detection (during meat inspection)	Personnel time (veterinarian)	No specific time allocated	37.96	0.534^2^	20.27
		Communication to LHU		30			
2	Data analysis and interpretation	Database investigation	Personnel time (veterinarian)	35	44.29	1	44.29
		Database updating and data transmission					
3	Farm survey (outbreak investigation) and samples collection	Round trip to the farm (standard cost)	Car usage	€ 20.00^3^	134.41	0.515^4^	69.22
			Personnel time (veterinarian)	30			
		Farm investigation and file compilation	Personnel time (veterinarian)	60			
		Drug prescription to farm dogs					
		Faecal sampling of farm dogs					
4	Laboratory analysis	Standard analytical procedure	All-embracing fixed cost	€ 58.87^5^	58.87	1.185 (0.515*2.3)^6^	69.76
Total					275.53		203.54

1 Minutes, if not otherwise indicated. Unit cost for one minute of LHU personnel (veterinarian) is 1.27 €_2019_, calculated on a one-hour standard cost of 74,00 € [22] and transformed into €_2019._

2 Rate of dairy cattle slaughtered in the Veneto region on total slaughtered (see S2 for details).

3 Official Veneto Region standard cost (€ 20.00) for LHA services car usage [22] and transformed into €_2019._

4 The rate is calculated considering the non-autochthonous cases (− 18.9%) according to [13] and the repeated cases, i.e. the cases coming from the same farm (− 36.5%) [13]. The rate is calculated thus as: (1–0.189)x(1–0.365) = 0.515 (51.5%).

5 Official standard costs applied to laboratory analyses by IZSVe (i.e. coprologic isolation of Taenidae - CPRTAE = 21.96 €; qualitative coprologic examination - CPRQL = 9.97; PCR + Sequencing of Cestoda - ARCEST = 26.94 €).

6 The rate is calculated multiplying the above rate for investigated farms by the average number of dogs found in a bovine farm [8].

The average cost for each bovine CE case resulting in a complete farm survey (including samples collection and analysis) was 275.43 €_2019_. In some cases, the procedure can be concluded at step 2 (Fig. 2 and Table 6), when the analysis of animal tracking suggests that the farm of origin found in Veneto cannot be the source of the infection. Therefore, considering the estimated implementation rate of each step (Table 6), an average cost of 203.54 €_2019_ was obtained. Considering that 101 cattle (95%CI: 87–122) are yearly notified to the veterinary services, the overall yearly costs for veterinary surveillance amount to 21,778.78 €_2019_ (lower estimate: 17,707.98 €_2019_; upper estimate: 24,831.88 €_2019_).

The overall cost for preventive treatment of dogs at risk for the two scenarios (appropriate protocol for all dogs at risk; prevention treatments actually in place) and parameters for their calculation are shown in Table 7. The unit cost of an appropriate yearly preventive treatment was considered to amount in at least 4 treatments a year for one dog and it was therefore estimated in 32.00 €_2019_. However, the current practice adopted in the Veneto region does not correspond to an appropriate preventive protocol. According to literature [8], 48% of sheep dogs were treated about two times per year, whereas the remaining ones were not treated or treated with unknown periodicity. These preventive treatments are paid by dog owners (private cost).

**Table 7:** Details of cost estimation for dog preventive treatments, in the two scenarios.

Description	Scenario	
	Appropriate prevention treatments protocol	Prevention treatments actually in place
Price (€/tablet)^1^	€ 4,00	€ 4,00
Average nr of tablets/dog	2	2
Nr admin/year	4	2

Cost of treatment of 1 dog/ year	32,00 €	16,00 €
Overall nr of dogs at risk	258	258
Percentage of treated dogs (among the ones at risk)	100%	48%

Overall cost for dog treatment/ year	€ 8.256,00	€ 1.981,44

1 Average price for 1 tablet of praziquantel-based drug, according to information provided by a retail franchise of pet supplies and available in the web.

The total yearly expenditure actually supported by sheepherders for dog treatments was estimated at 1981.44 €_2019_, but an appropriate preventive protocol for the whole sheep dog population would imply a yearly cost of 8256.00 €_2019_.

### 3.4 Data synthesis

**Fig. 3:**
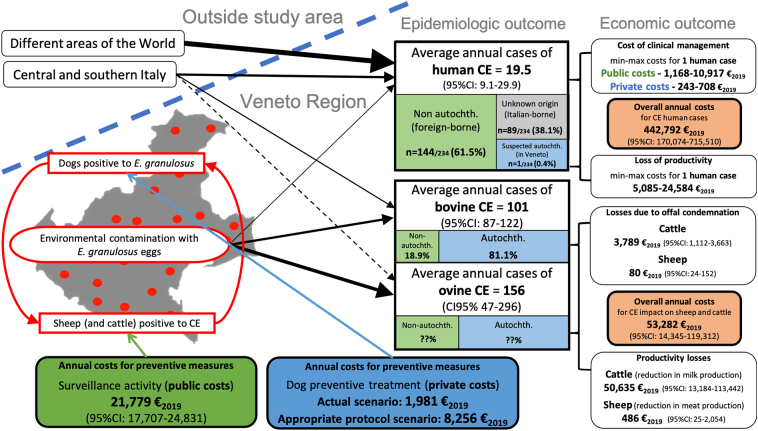
Flow chart reporting the main epidemiological and economic outcomes of CE in the Veneto region, on an annual basis. Note: The black arrows represent the causative flows, and their size is proportional to the relative importance of each flow; the dotted line indicates an undemonstrated causative flow. Coloured lines link each specific preventive measure to the stage of the parasite life cycle targeted by the intervention.

The overall annual economic impact of CE in the study area was estimated at an average of 496,074.93 €_2019_ (95%CI: 184,419.77-834,822.29 €_2019_). Fig. 3 depicts the flow chart developed during the first phase of the project [10], filled with the numbers retrieved during the second phase, and summarizing the epidemiological and economic outcomes of CE in the study area.

As highlighted in Fig. 3, the economic impact of the relatively few human cases is about eightfold higher than the costs related to the animal component, which are mainly due to the reduced milk production by infected dairy cows. The preventive measures in place at present in the region account for about 24,000.00 €, mostly attributable to the veterinary surveillance activity.

## 4 Discussion

The findings of the present study allow for an improved understanding of the impact of CE in the study area, laying the foundations for an evidence-based decision-making process on the measures to control this disease. The analysis of the HDRs showed an estimated incidence rate in the human population of 0.4/100,000 people per year, which is in line with what already reported for northern Italy and for other hypo-endemic regions in  wealthy and industrialized economies [7,9,25]. More importantly, our findings suggested that the source of the infection for most of the human cases is external to the study area, although few cases were possibly autochthonous. Concerning animals, this study attempted for the first time to estimate the prevalence of CE in sheep in the study area (estimated at 6.0%). Prevalence estimation in sheep based on slaughterhouse records are difficult to achieve in Italy, due to the lack of an efficient data flow from slaughterhouse to LHAs. Our finding is slightly exceeding the value predicted for sheep farm prevalence in the Veneto region in a recent study [26], which was however including also young animals in the denominator, among slaughtered sheep. The calculated prevalence value for CE in cattle (0.18%) is in line with values reported in other regions of Northern Italy [27,28].

The overall economic impact of CE in the region is less than 0.5 million €_2019_, as expected in a hypo-endemic area. A similar total cost (483.608,00 €) was estimated in the year 2004 for Lombardy (8,410,374 inhabitants), a region neighbouring Veneto [28]. However, that study considered only hospitalization costs for human cases. There are many studies on the economic impact of CE in developing countries with low-middle income [[29], [30], [31], [32]], which are generally estimating an overall yearly cost of a few millions US$ for entire countries, such as Jordan and Peru, or a few hundred thousand US$ for areas with smaller populations, such as the Tibetan Plateau. Only a few studies were conducted in regions or countries with socio-economic conditions similar to our study area. In Spain, an overall economic impact of about 150 million € was estimated in 2005 [33], meaning a monetary estimation 300 times higher than that obtained in the present study. This difference depends on the different size of the considered study areas (about 43 million inhabitants in Spain in 2005 vs 5 million in Veneto) and can be partly due to the higher incidence of hospitalized human CE cases recorded in most regions of Spain [7], compared to Veneto. Besides, this study included different costs (reduction in carcass weight, reduction in milk production and decrease in fecundity) associated to four domestic species (sheep, goats, cattle, pigs). In Wales (2,900,000 inhabitants), the total annual cost due to CE in 1997 was estimated to range from about 1000,000 US$ to about 6,700,000 US$ [25], which is anyhow proportionally higher than our estimation for Veneto. One important difference for the case of Wales lies on the higher contribution provided by animal-health costs that are more than threefold higher than human-health costs. In this study, reduction in carcass and fleece values and reduction in fecundity associated with sheep were considered. According to our results, on the contrary, the relatively few human cases occurring in residents in Veneto imply an important cost (about 443,000 €/year) for the regional health system and the overall economy of the Region. The impact of the disease in animals instead was not very relevant (about 53,000 €/year), and mostly due to reduced milk production in cattle. The human:animal costs ratio is about 8:1, which is therefore the opposite to what found in Wales [25], but similar to Spain [33].

Our study highlighted a number of limitations in the attempt to achieve a detailed estimation of the economic impact of the disease. Firstly, non-hospitalized human CE cases not captured by the HDR system were not retrievable. Therefore, the number of human cases medically treated or managed by watch-and-wait (patients with asymptomatic, inactive CE undergoing only regular control with imaging), and consequently the derived costs estimations, are underestimated. Also, due to the rarity of life-long disability caused by CE, for example in case of osseous involvement, and the absence of such cases in the examined dataset, this instance was not considered in our EEM. Finally, we could not estimate, and therefore quantify costs related to the proportion of infected patients with some disability due to symptoms who are not diagnosed (as having CE or at all).

The lack of active surveillance and control activities for the human component complicated the estimation of epidemiological and economic outcomes. However, this is partly justified by the nature of the disease in humans, who are a dead-end host for the parasite. Therefore, human case management does not impact parasite transmission cycle to either animals or humans; that is, does not prevent further human cases. Early diagnosis and treatment of human CE, ideally achieved through active surveillance, may reduce the cost of infection, as more cases could be managed by medical therapy only. However, active surveillance of CE cannot be implemented through serology [34], and regular screening by imaging (abdominal ultrasound and chest X-ray) should be carefully evaluated in the light of cost-benefit analyses in an area of very low endemicity and compliance with principles for disease screening [35].

Finally, although we elaborated a case definition to identify autochthonous cases, the origin of the majority of the human infections remains uncertain. Most cases were probably imported, and only one human case (0.4%) in the whole observation period was strongly suspected to be autochthonous. The identification of the autochthonous nature of human cases can be attempted, if at all, only at the time of the anamnestic interview and therefore clinicians should receive appropriate training on this emerging infection, including a knowledge update on the local epidemiology of *E. granulosus s.l.* in animals. However, an accurate epidemiological tracking of the source of human infections, similar to what implemented in livestock farms, is almost always impossible because no acute signs of infection occur, and the vast majority of human CE cases have a slow cyst development and remain asymptomatic for a very long time.

As per Directive 2003/99/EC on the monitoring of zoonosis and zoonotic agents, regional and local health authorities built up a quite complex monitoring system for bovine CE, targeting the infected intermediate animal hosts and aimed at identifying the source of the infection. These preventive measures account for a cost of about 22,000 €_2019_ per year; however, their efficacy in controlling the disease is under question and a new approach to the management of CE cases in cattle was already proposed, since the bovine farm dogs were very rarely found to be the source of the infection [8]. On the contrary, the tracking system for sheep CE appeared to be inefficient, and there was a general lack of data on sheep infection at slaughterhouse. In fact, most LHAs declared that they did not receive any notification of CE from slaughterhouses, notwithstanding the 6.0% prevalence in sheep reported directly by slaughterhouse officials in response to our active data request. Furthermore, this data relies on the answer received by only part of the slaughtering facilities actively contacted (63.6%) and therefore the estimated infection rate may suffer of a bias due to this. The surveillance system in animals and more specifically in sheep needs therefore an improvement, and data reporting to LHAs has to be uniformly assured by all slaughterhouses in and outside the Veneto region.

Preventive measures implemented by private citizens (treatment of sheep dogs) accounted for a limited cost (about 2000 € per year). However, the treatment protocols applied by shepherds proved to be sub-optimal, consisting of an average of two administrations per year (or less), while the recommended protocol should ideally rely on one treatment every six weeks, and realistically aims for four treatments per year. A public support to cover the cost of the drugs for sheepherders can account for about 8500 €/year. However, this action needs to be associated to the implementation of specific training for sheepherders and a new system to monitor the regular treatment of sheep dogs.

Finally, although the picture drew in the present study allowed for an overall estimation of the importance of CE in the study area, our EEM did not consider the contribution that could be provided by other domestic (e.g., goats, pigs) and wild (e.g., wolf, wild ruminants) animals. Their role was considered negligible during the building of the EEM [10]; however, this situation may change in the future, considering the dynamic nature of many ecological and social phenomena (e.g. the epidemiological role of the wolf can increase in the future, in consideration of his recent colonisation of the study area).

## 5 Conclusion

CE annual economic impact in the Veneto region is mostly due to human-health costs and amounts to about 0.5 million €_2019_. The economic impact of the relatively few human cases is about sevenfold higher than the costs related to the animal component, which are mainly due to the reduced milk production in infected dairy cows. The source of most of the human cases was likely external to the study area and this finding suggests that locally implemented preventive actions have a limited impact on the reduction of the burden of the disease. However, considering that a surgically treated case can account for an overall cost exceeding 30,000 €, even few autochthonous cases can imply an annual cost for the regional economy that is by far exceeding the overall yearly cost of preventive actions on animals. Thus, an improvement in the veterinary surveillance system (including the data flow management) can allow the early identification of emerging clusters of local parasite circulation, preventing the risk for an increment in the number of autochthonous human cases [36,37].

## Funding

This study was supported by the project “10.13039/501100009878Evaluation of policy measures to control two emerging parasitic diseases (Cystic Echinococcosis and Leishmaniasis) in Veneto Region using a One Health approach” (cod. BIRD174940), granted in the framework of the Departmental integrated research budget of the University of Padova, year 2017.

## Declaration of Competing Interest

On behalf of all co-authors (Massimo Canali, Francesca Tamarozzi, Andrea Angheben, Gioia Capelli, Federico Gobbi, Matteo Legnardi, Michele Brichese, Giuseppina Napoletano, Fabrizio Cestaro, Adriano Casulli, Michele Drigo, Maurizio Aragrande) I hereby state that we have no conflicts of interest to disclose concerning the manuscript “A One-Health evaluation of the burden of cystic echinococcosis and its prevention costs: case study from a hypo-endemic area in Italy”.

## Appendix A Supplementary data

Supplementary material S1 HDRs human echinococcosis Veneto. Image 1

Supplementary material S2 Human costs. Image 2

Supplementary material S3 Animal costs. Image 3
